# Minimally invasive colonoscopy treatment of inflammatory fibroid polyps in the terminal ileum

**DOI:** 10.1038/s41598-023-31719-0

**Published:** 2023-03-26

**Authors:** Yin-Si Tang, Lu Liu, Ying Gao, Qiao-Chu He, Hai-Mei Guo, Zhi-Feng Zhao

**Affiliations:** 1 Department of Gastrointestinal Endoscopy, The Fourth Affiliated Hospital of China Medical University, No.4 Chongshandong Road, Huanggu District, Shenyang, 110032 China; 2 Department of Pathology, The Fourth Affiliated Hospital of China Medical University, No.4 Chongshandong Road, Huanggu District, Shenyang, 110032 China

**Keywords:** Cancer, Cancer therapy, Gastrointestinal cancer

## Abstract

To identify the characteristics of inflammatory fibroid polyps (IFP) in the terminal ileum and to investigate the methods, safety, and efficacy of colonoscopic minimally invasive dissection and resection therapies for its treatment. Colonoscopy and colonoscopic ultrasonography were used to diagnose patients with protruding mucosal lesions in the terminal ileum, and the results suggested a high suspicion of IFPs. Colonoscopic minimally invasive dissection and resection were performed for these patients, and IFP was confirmed by postoperative pathological examination and immunohistochemical staining. Twelve cases of IFP from April 2016 to December 2020 in our hospital were examined pathologically and immunohistochemically. The IFPs in the terminal ileum were all successfully excised by colonoscopy. There were no postoperative perforation, bleeding, or recurrence cases during the follow-up. The features of the lesions, as well as the efficacy of colonoscopic minimally invasive dissection and resection, were reviewed. Terminal ileum IFPs have corresponding colonoscopic and endoscopic ultrasonographic features. For IFPs less than 2 cm in size and within 10 cm of the ileocecal valve, removal by colonoscopy was proven less invasive, safe, and effective.

## Introduction

Inflammatory fibroid polyps (IFP), also known as “Vanek tumors”, are rare interstitial lesions of the digestive tract^1^. The lesion was first reported in 1949 and was called “submucosal granuloma with eosinophilic infiltration”, which was later renamed “inflammatory fibroid polyps” in 1953^2,3^. IFPs tend to occur in adults aged 20 to 90, with an average age of 60. Its typical size ranges from 0.2 to 4.2 cm, with an average of 1.7 cm. IFPs are usually located in the stomach and small intestine, and the incidence is slightly higher in women. The clinical symptoms are related to the location and size of the lesion. IFPs in the gastrointestinal tract can manifest with weight loss, melena, nausea, and abdominal pain, and patients with large lesions are prone to intussusception and intestinal obstruction. Many patients are diagnosed by incidental symptomatic examination^1,4^. Small intestinal IFPs often grow in the terminal ileum. Due to its rarity^5^, clinicians often misdiagnose IFP, especially in the terminal ileum, which is usually small and without corresponding clinical symptoms.

Currently, there is no relevant literature or reviews on minimally invasive resection of terminal ileum IFP via colonoscopy. From April 2016 to December 2020, 12 cases of terminal ileum IFP (confirmed by postoperative pathological and immunohistochemical examinations) were successfully resected in our Digestive Endoscopy Center. The clinical characteristics of the above cases were retrospectively analyzed and summarized.

## Results

### Clinical characteristics

A total of 12 cases of terminal ileum IFPs were diagnosed and resected successfully, including 4 males and 8 females. Notably, 8 out of 12 patients were female (66.7%, 8/12), which is consistent with the incidence of IFPs. The mean age was 59.4 ± 10.87 years. Two patients had recurrent pain in the lower right abdomen, one had positive fecal occult blood, and the other 9 showed no clinical manifestations (Table 1).

**Table 1:** Clinical characteristics of cases.

Seq.no	Age	Sex	Size (cm)	Surface mucosal characteristics	Clinical symptoms	Ultrasound characteristics	Duration (min)	Complications	follow-up
1	50	Female	0.8	Congestion and erosion	None	Hypoecho of submucosal origin	85	None	Not followed up
2	54	Female	1.5	Villous structure	None	Hypoecho of submucosal origin	107	None	No recurrence
3	49	Male	0.8	Villous structure	None	Hypoecho of submucosal origin	104	None	No recurrence
4	68	Male	1	Villous structure	Abdominal pain	Hypoecho of submucosal origin	96	None	Not followed up
5	42	Female	1.2	Congestion and erosion	None	Hypoecho of mucosal muscular origin	127	Fever	No recurrence
6	51	Female	0.8	Villous structure	None	Hypoecho of submucosal origin	113	None	No recurrence
7	68	Female	1	Hyperplasia-like changes	None	Hypoecho of submucosal origin	49	None	No recurrence
8	70	Female	1.8	Villous structure	Abdominal pain	Hypoecho of submucosal origin	67	None	No recurrence
9	62	Male	0.5	Villous structure	None	Hypoecho of mucosal muscular origin	34	None	No recurrence
10	78	Female	1.2	Villous structure	None	Hypoecho of submucosal origin	38	None	Not followed-up
11	54	Male	0.5	Congestion and erosion	None	Hypoecho of submucosal origin	55	None	No recurrence
12	67	Female	1.5	Hyperplasia-like changes	Fecal occult blood positive	Hypoecho of submucosal origin	102	None	Not followed-up

### The colonoscopic findings

**Figure 1:**
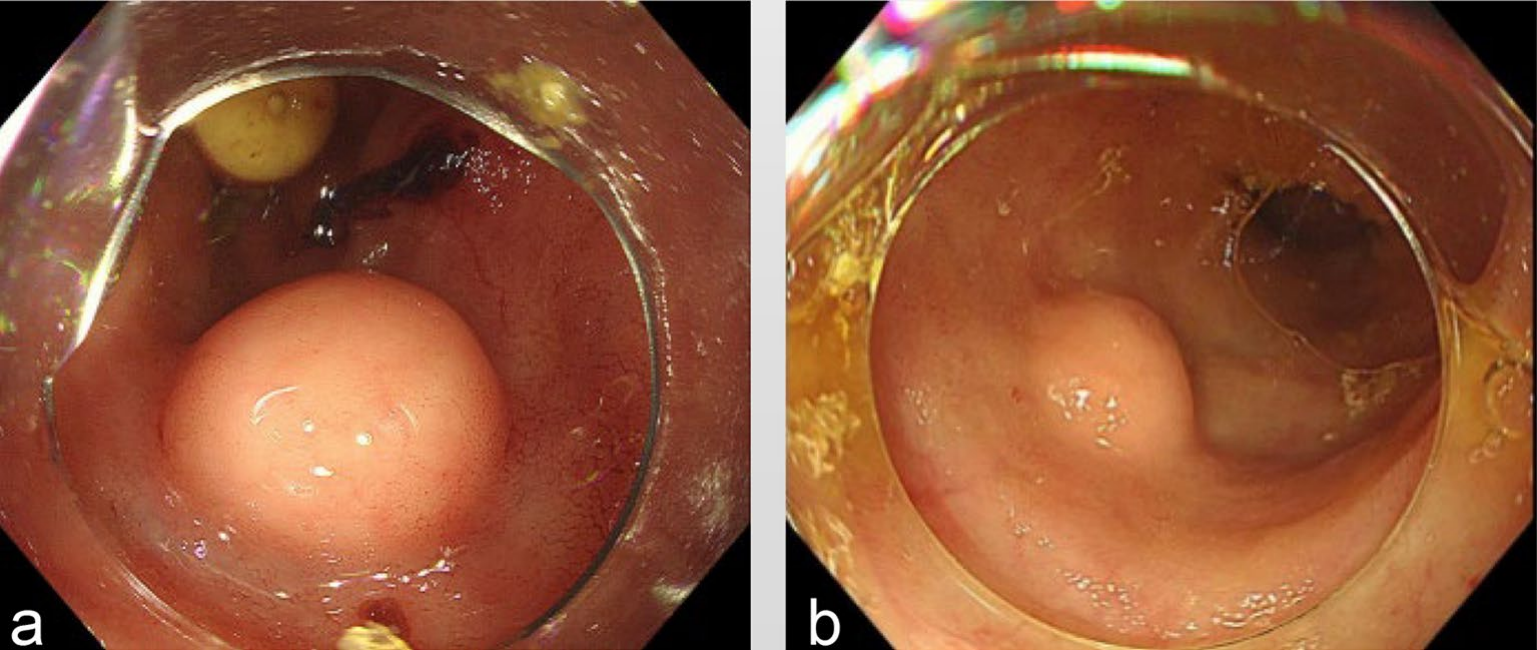
White Light Endoscopes: The mucosa is protruded, with a smooth, slightly yellow surface. (**a**) The mucosa showed bubble-like protuberance. (**b**) The mucosa showed mound-like protuberance.

All cases were examined with a white light endoscope and had solitary lesions. The lesions were round, hard, and yellowish without deformation, had a rough texture, and were fixed in position 2–8 cm away from the ileocecal valve. Nine cases had mound-like protuberances, while the other 3 cases had bubble-like bulges (Fig. 1). The surface mucosa showed normal villous-like structures in 7 cases, hyperplastic changes in 2 cases, and congestion and erosion in 3 cases. Due to the submucosal origin of the lesions, mucosal surface changes were considered related to chronic inflammatory irritation (Table 1). The 12 cases can be classified into two types based on their morphology during colonoscopy: “mound” and “bubble”. The mound type showed a mound protuberance with an obtuse intersection angle between the lesion and normal mucosa and a surface mucosa consistent with normal ileal mucosa (Fig. 1a). The bubble type had a submucosal bubble-like protuberance with an intersection angle between the lesion and normal mucosa and a slightly thin surface mucosa prone to erosion (Fig. 1b). In our study, the majority of cases had mound lesions (9/12).

### Colonoscopic ultrasound findings

**Figure 2:**
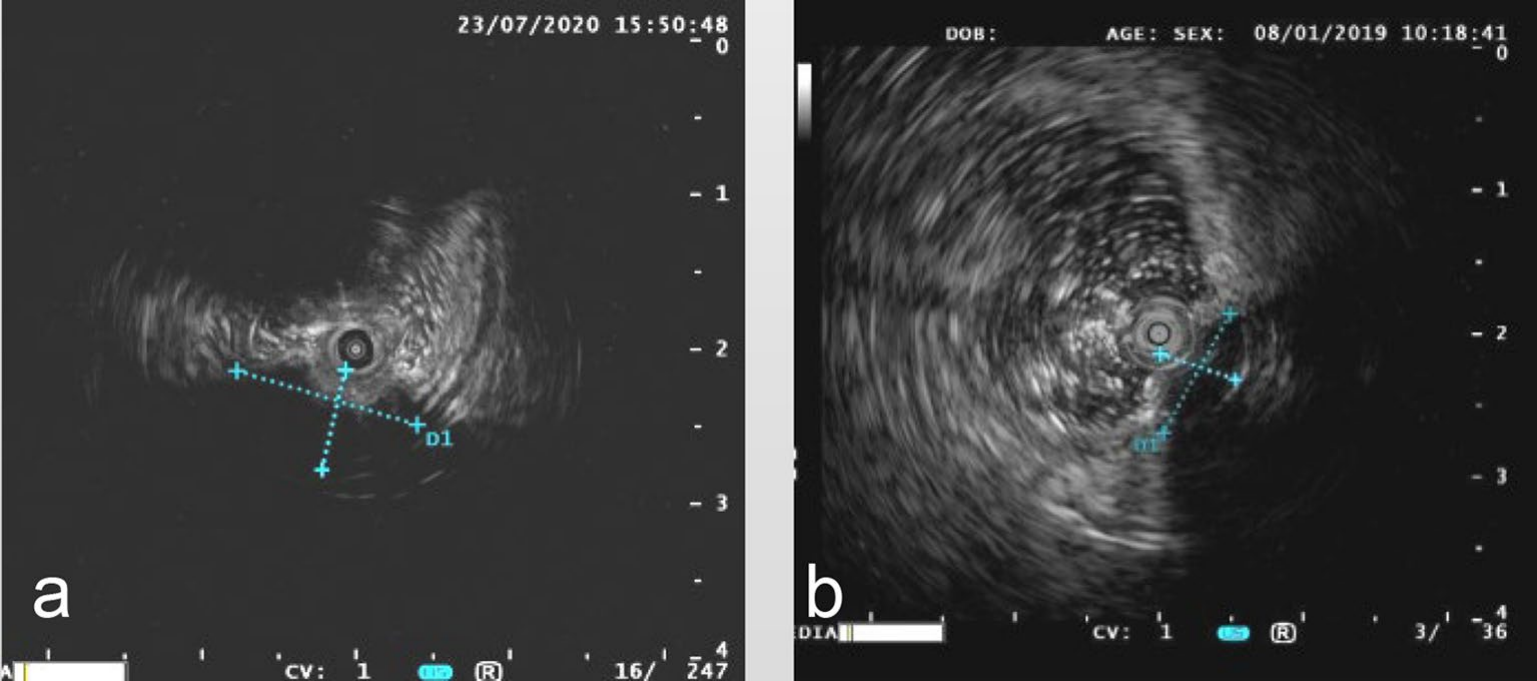
Colonoscopic ultrasound. (**a**) A hypoechoic mass is observed in the submucosa, which was demarcated from the muscularis propria. (**b**) A hypoechoic mass in the submucosa that is poorly demarcated from the muscularis propria.

A preoperative ultrasound colonoscopy was performed in all cases. The mean lesion size was 1.05 ± 0.41 cm. The lesions originated from the muscularis mucosa in 7 cases and from the submucosa in 5 cases, of which 3 cases were poorly demarcated from the muscularis propria, and the remaining 2 cases were well-delineated. In all cases, the lesions grew into the cavity and had homogeneous internal echotexture equal to or slightly higher than the muscle echotexture (Fig. 2). Colonoscopic ultrasonography of “mound” lesions demonstrated poor demarcation between the lesion and the muscularis propria. Colonoscopic ultrasonography of “bubble-type” lesions revealed that the lesions were demarcated from the muscularis propria. Therefore, “bubble-type” lesions were more easily dissected via colonoscopy.

### The surgical procedure

**Figure 3:**
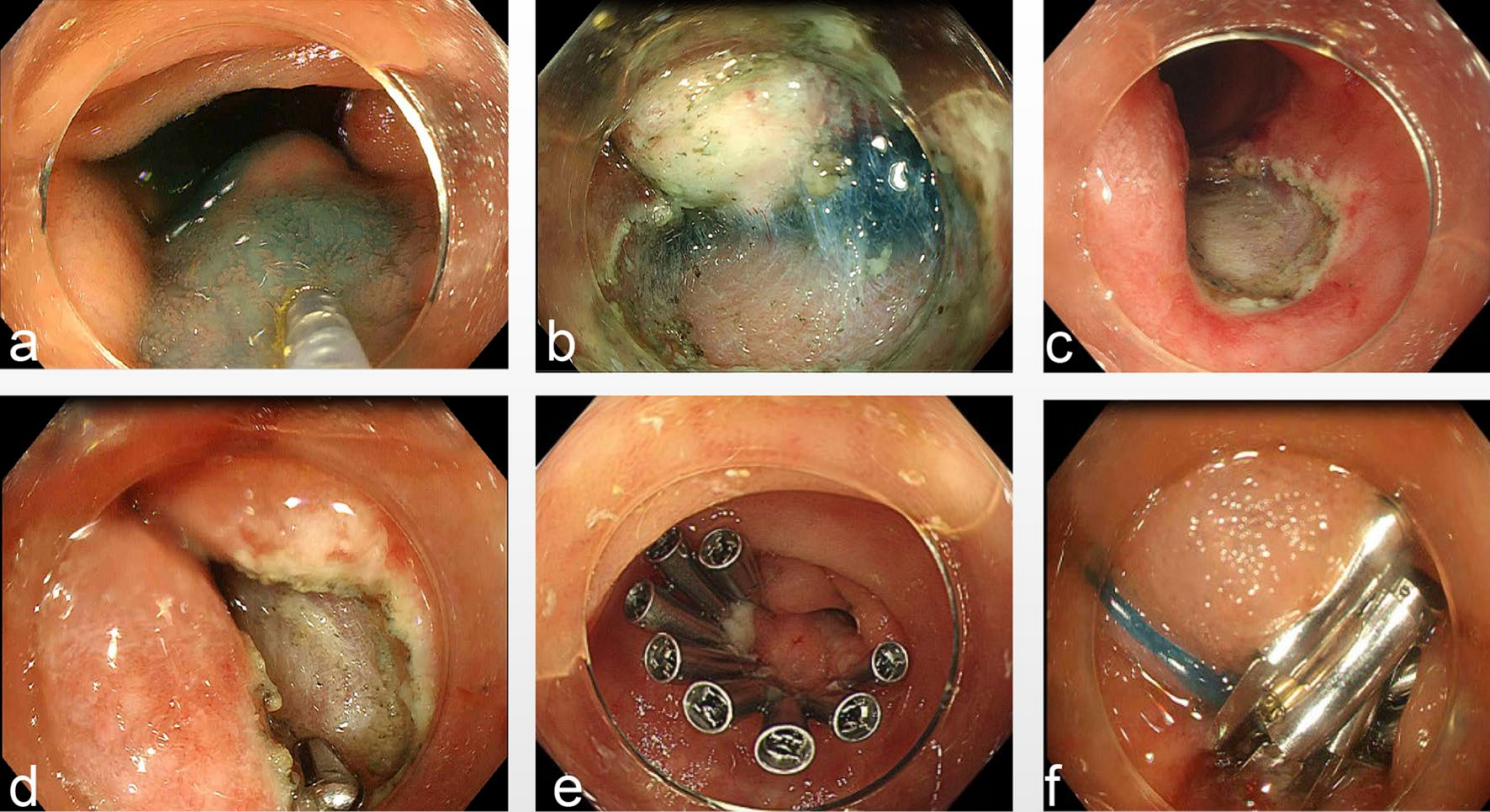
(**a**) Submucosal injection. (**b**) Dissection of the lesion along the muscularis propria. (**c**) Hemostasis of the wound surface using heat forceps. (**d**) The muscle layer of the wound surface was intact after the operation. (**e**) The wound was closed by a hemostatic clip. (**f**) Hemostatic clip-assisted nylon suturing for the wound.

Colonoscopic mucosal dissection and resection were performed in all cases, and 3 cases required snare-assisted mucosal dissection and resection. There were no residual lesions in any of the cases, and the success rate of resection was 100%. One patient was switched to endotracheal intubation and general anesthesia during surgery due to excessive respiratory amplitude, which affected the colonoscopic procedures. The mean surgical duration was 81.42 ± 31.60 min. Three cases had poorly elevated lesions after submucosal injection with unclear submucosal levels, all of which were “mound type” cases, the demarcation between the lesion and the internal surface of the muscularis propria was unclear during the dissection process. This is consistent with what has been described by preprocedural endoscopic ultrasonography;. In contrast, cases of the “bubble type” generally have a distinct elevation sign, clear submucosa, and relatively easy dissection. When the submucosal space was unclear, electrocoagulation and resection were applied along the surface of the tumor using a mucosa-cutting knife. Two cases had intraoperative bleeding. Both cases were “mound type” cases, with relatively rich vessels and poor vascular exposure due to poor visualization in the submucosal space, but both cases were successfully hemostatic by thermocoagulation forceps. A single case had a muscular layer injury during the procedure, and a hemostatic clip and nylon purse-string suture were used. Direct closure with hemostatic clips was attempted in the other 11 cases, 10 of which were successful; one case was difficult due to a large wound surface, and an additional purse-string suture was used. No indwelling drainage tube was used in any patient (Fig. 3).

### Pathological and histochemical findings

**Figure 4:**
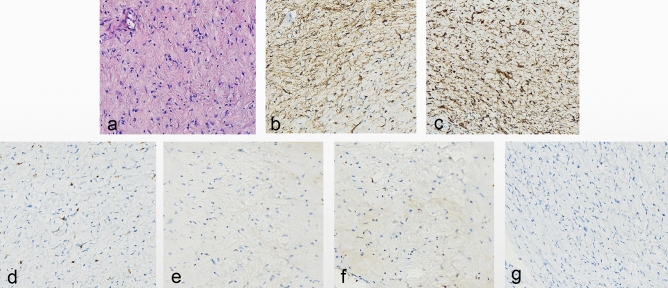
(**a**) H&E staining showed spindle cell proliferation and onion peel-like structures surrounding the blood vessels in a whorled pattern (X20). (**b**) CD34 positive expression (X20). (**c**) Vimentin positive expression (X20). (**d–f**) CD117/DOD-1/s-100 was not expressed (X20). (**g**) The Ki-67 proliferation index was low (X20).

IFPs lesions are located below the mucosa, which is a histopathological feature of IFPs, making it difficult to perform a biopsy to confirm the diagnosis. All 12 patients in this study had postoperative pathology examinations to confirm the diagnosis, which was consistent with the characteristic features of IFPs. Spindle cell proliferation was observed, and onion peel-like structures surrounded the blood vessels in a whorled pattern with multiple eosinophils and inflammatory cell infiltration. Immunohistochemistry was conducted in all 12 cases, and the results showed vimentin (+), CD117 (−), DOG-1 (−), S-100 ( −), desmin (−), and low Ki-67 proliferation index (1–5%). Eleven cases were CD34 (+), while one was CD34 (partially+) (Fig. 4). The incidence of IFPs at this site is significantly higher than other spindle-cell tumors, such as leiomyomas and stromal tumors.

### Postoperative complications and follow-up

In one case, the anesthesia method was switched to endotracheal intubation. During this patient’s procedure, the demarcation between the lesion and the muscularis propria was not clear, the submucosal space was not obvious, and electrocoagulation dissection was carried out between the lesion and the muscular layer, with a probability of muscularis injury. This patient developed a fever 24 h after the procedure, and routine blood tests revealed a bacterial infection. Relevant tests excluded infections at other sites, and levofloxacin was administered intravenously. The patient’s body temperature dropped the next day, and normal blood test parameters were restored. All patients had no severe myometrial injuries, late postoperative bleeding, perforation, or postoperative intestinal luminal narrowing. The hemoclip in one patient was removed by colonoscopy 1 month after the operation. Eight cases showed no recurrence through colonoscopy at 1 month, 3 months, and 1-year follow-ups after surgery, two cases did not continue colonoscopic follow-up, so there is no colonoscopic follow-up data available for them as of now. Findings from follow-up visits suggested that IFPs are solitary lesions with no postoperative recurrence.

## Discussion

### The current treatment of IFP in literature

PubMed was searched for relevant studies using the terms “inflammatory fibroid polyp” and “Vanek tumor”, and a total of 496 articles were retrieved. There were 378 articles related to IFP treatment and 165 about IFP endoscopic treatment. Among the endoscopic treatment articles, there were 84 articles about intragastric lesions, 36 on colonic lesions, and 1 on an enterostomy for distal ileal lesions^6^. Terminal ileum IFPs are rare, and the symptoms and signs of small intestinal diseases are generally indistinct due to their position in the middle of the digestive tract, so the diagnosis is often missed in clinical practice. Currently, double-balloon enterostomy and capsule endoscopy are ideal examination methods^7^. However, they are not commonly used in hospitals. Instead, small intestinal diseases are often diagnosed using barium small bowel follow-through in primary hospitals. With the advancement of colonoscopy technology, terminal ileum examination using colonoscopy will make it easier to early detect and treat IFPs. Studies have also indicated that colonoscopy treatment of terminal ileum lesions is equally safe and effective^8–11^.

### The origin of IFPs and the necessity of their resection

To this day, the etiology of IFPs remains unclear, though many studies have suggested that they may be related to chemical, physical, and metabolic inducing factors^5^. IFP gene studies have indicated an IFP association with platelet-derived growth factor receptor alpha (PDGFRA) mutations^12^, which are also seen in KIT-negative gastrointestinal stromal tumors. Therefore, some researchers have proposed that IFPs may be neoplastic rather than a simple inflammatory lesion^13^. Most researchers believe that IFPs are not metastatic or aggressive, and their onset is insidious. However, some lesions may gradually grow in size and cause complications, including intussusception^14^, abdominal pain^15,16^, bleeding^17,18^, and obstruction^19,20^, so resection is recommended.

### The importance of insertion rate in the terminal ileum during colonoscopy

All lesions in this study were small in size and resected early in the course of the disease using colonoscopic resection. The lesions were mainly early-onset and without complications. The insertion rate of the terminal ileum by colonoscopy in our gastrointestinal endoscopy clinic is about 86.5% (4439/5132), and the detection rate of end ileal disease is 8.8% (392/4439). We retrospectively analyzed 12 treated cases of terminal ileum IFPs from 2016 to 2020 in our hospital, accounting for 3.1% (12/392) of all lesions in the terminal ileum. Therefore, the insertion rate of the terminal ileum is emphasized during colonoscopy to improve the detection rate of terminal ileum lesions, which contributes to the early diagnosis and treatment of diseases in the terminal ileum. There was some inconsistency between the clinical characteristics of the present study and previous observations, which could be attributed to the small sample size.

### Surgical challenges and procedural skills

During the colonoscopic procedure, there were some technical challenges and corresponding procedural skills: (1) The lesion was close to the ileocecal valve, so the colonoscopy was liable to slip out the ileocecal valve, and it was necessary to repeatedly enter the ileocecal valve during the colonoscopic operation, and the operator must be skilled at controlling the colonoscope. In this situation, the operator should try to make the endoscopy body form a fulcrum outside on the examination bed to stabilize the endoscopy body. To ensure the accuracy of the operation, the operator should handle the endoscopy body alone, and the assistant should not fix the endoscopy body. (2) The intestinal wall of the terminal ileum is thin, the submucosal layer is weak, and the submucosal injection layers are not well displayed, so the possibility of damaging or perforating the muscularis propria or the tumor is high. Thus, carbon dioxide was used during the procedure. The injection points were selected 0.5 cm away from the lesion, allowing the submucosal injection fluid to gradually extend to the lesion. Then the mucosa was incised to establish the submucosal space. (3) Endoscopy progression is difficult in the terminal ileum, where it is difficult for the colonoscope to access the lesion, or the transparent cap is difficult to function. This difficulty is usually associated with failure to straighten the endoscopy body or excessive insufflation during manipulation. Thus, the transparent cap must be fully applied to progress the colonoscope along the lumen, reduce the injection of CO2, untie the formed loop in time, and ensure that the colonoscope reaches the ileocecal junction without loop formation. Colonoscopic treatment at this site requires a high level of technical experience; therefore, the director of the Department of Gastrointestinal Endoscopy at our hospital operated on the 12 cases. He completed more than 6000 cases of endoscopic mucosal dissection (ESD). Traction devices were not used in any of the 12 cases. However, for relatively less experienced operators, using appropriate traction techniques can help expose the lesion more fully and facilitate the establishment of the submucosal space, thereby reducing the difficulty of the procedure. (4) Colonoscopic suturing of lesions at this site was difficult, but the operator was experienced, and all wounds were successfully sutured with hemoclips or nylon sutures. For less experienced operator, the insufflation should be reduced to ensure better visualization during the procedure, and the transparent cap should be used more frequently to expose the surgical field. Less insufflation can help to avoid overstretching of the intestinal canal, thereby reducing the difficulty of suturing.

ESD at the terminal ileum requires a very experienced operator to perform it, and for less experienced operators, resection of less complexed lesions can be attempted based on the above skills.

### Pathological characteristics of IFPs

The main histopathological feature of IFPs is the lesions’ location below the mucosa. They present as short spindle-spindle cells arranged in bundles or sheets with medium cell density and an abundance of inflammatory cells in the background. Eosinophils in varying amounts are also commonly found in the interstitium^4^.

The white-light colonoscopy and endoscopic ultrasonography findings of terminal ileum IFPs must be differentiated from lipomas. Lipoma is soft at white-light colonoscopy, without a “bubble” appearance, while under ultrasound colonoscopy, a lipoma is manifested as a homogeneous hyperechoic mass of submucosal origin demarcated from the muscularis propria^21^.

Additionally, IFPs must be histopathologically distinguished from some digestive tract tumors, non-neoplastic diseases, and spindle cell lesions, including inflammatory fibrosarcoma, spindle cell carcinoid, leiomyoma, gastrointestinal stromal tumor, and nerve sheath tumor^4,5^. All lesions in the present study were immunohistochemically examined after resection^4^. The immunohistochemical results of all 12 lesions showed that CD34 and vimentin were highly expressed, while CD117 was not expressed. Therefore, the incidence of IFPs at this site was considered significantly higher than that of other spindle cell tumors, such as leiomyomas and stromal tumors.

## Conclusion

IFPs have a fixed spherical protuberance of the mucosa with a slightly yellowish color and hard texture in the terminal ileum. Colonoscopic ultrasound showed that IFPs originate from the inner surface of muscularis propria, protrude into the cavity with a clear boundary, and have equal or slightly higher echogenicity than muscle. Colonoscopic minimally invasive dissection and resection is an important method for treating terminal ileum IFPs, with advantages in terms of safety, efficacy, and reduced trauma. Colonoscopy-assisted removal of IFPs less than 2 cm in size and within 10 cm proximal to the ileocecal valve is safe and effective.

## Methods

The ethics committee of our institution has issued ethical approval for this study.

### Case selection and inclusion criteria

Records of colonoscopy cases in the Fourth Hospital of China Medical University between April 2016 and July 2020 that met the following criteria were reviewed: (1) Colonoscopy revealed protruding lesions in the terminal ileum; (2) the lesions were consistent with IFPs based on colonoscopic ultrasonography; (3) mucosal dissection and resection were conducted; and (4) postoperative pathological and immunohistochemical examinations were consistent with IFP. A total of 12 patients met the above conditions, and informed consent was obtained from all of them. Colonoscopic ultrasonography and colonoscopic minimally invasive resection were performed by experienced specialists in all cases.

### Preoperative preparation and colonoscopy

Routine blood tests, coagulation tests, blood gas tests, pulmonary function tests, Doppler echocardiography, and other preoperative examinations were carried out 24 h before surgery. Dimethicone was also orally administered 6 h before surgery after bowel preparation using polyethylene glycol electrolyte solution. Intravenous general anesthesia was used. Cap-assisted colonoscopy was progressed to the ileocecal junction using a distal attachment cap (XT-DL-128-40, Shangxian, China) attached to the distal end of the electronic colonoscope (CF-HQ290AI, Olympus, Japan). The length of the endoscopy progression was recorded as the colonoscope continued to advance into the terminal ileum for at least 10 cm to observe the morphology, size, texture, and surface mucosa of the lesion.

### Ultrasound colonoscopy

Using the degassed water congestion method, a miniature ultrasound probe (UM-2R/UM-G20-29R, Olympus, Japan) was used to scan the lesion at 2.0–3.0 cm from the lesion at an ultrasound frequency of 20 MHZ. The lesion’s size, origin, border, echogenicity, and infiltration were recorded.

### Colonoscopic resection method

Racemic anisodamine hydrochloride was given intramuscularly before the procedure to reduce intestinal peristalsis. CO2 insufflation was used during colonoscopies, and normal saline containing methylene blue, Hemocoagulase Bothrops Atrox, and hyaluronic acid (ratio: 40 ml 0.9% normal saline + 8 KU Hemocoagulase Bothrops Atrox + 6 ml sodium hyaluronate + 1.1 ml methylene blue injection) was injected using an injection needle (AMH-SYB-2418-2304, Anrui Medical, China) to the submucosa under the lesion. The distal mucosa of the lesion was incised using a mucosa-cutting knife (AMH-EK-O-2.4X2300(4)-N, Anrui Medical, China), creating the submucosal space, gradually separating the tumor and the muscularis propria, and expanding the incision to both sides of the lesion until the tumor was fully exposed. During the operation, pre-hemostasis and hemostatic treatment were performed on submucosal vessels using thermal forceps (AMH-HF-A-2.4X2300, Anrui Medical, China). A mucosa-cutting knife was used to maximize the separation of the tumor and the muscularis propria, and a snare (AMH-SNER241524, Anrui Medical, China) was then applied to remove the lesion whenever it was difficult to dissect the tumor completely. The ERBE VIO 200D generator (ERBE, Germany) was selected for the surgery. After surgery, hemostatic clamps (AMH-HCG-195-135, Anrui Medical, China) were routinely applied to close the wound. For difficulties in suturing the wound surface or suspected muscular layer wounds, a ligation device (LD-230, LeoMed, China), nylon suture (Loop 20/30, LeoMed, China), and hemostatic clip were used for purse-string suturing (Video 1).

### Pathological and immunohistochemical examination

The specimens were preserved in 10% formalin, routinely embedded in paraffin, sectioned, stained with H&E, and identified by immunohistochemistry. Immunohistochemistry included CD34, CD117, vimentin, DOG-1, S-100, desmin, actin (SM), and Ki-67. The diagnosis was based on the second edition of “Biopsy Interpretation of the Gastrointestinal Tract Mucosa” by Elizabeth A. Montgomery.

### Postoperative treatment and follow-up

The patients were prescribed postoperative fasting, absolute bed rest, acid suppression, fluid replacement, and hemorrhage prevention treatment. A routine blood test was performed on the second day after the operation. Soft food without residue was permitted after 48–72 h, with a gradual transition to a regular diet. Fasting time was extended for patients who had intraoperative injuries or large wound surfaces. Colonoscopy was repeated 1 month, 3 months, and 1 year after surgery to assess wound healing and check for residual hemostatic clips or recurrence.

### Ethical statement

All methods were carried out in accordance with the Declaration of Helsinki and the regulations of our hospital.

All experimental protocols were approved by the IRB of the Fourth Affiliated Hospital of China Medical University.

Informed consent was obtained from all patients who agreed to publish this article.

## Supplementary Information

Supplementary Legends.

Supplementary Video 1.

## Data Availability

The datasets used and/or analyzed during the current study are available from the corresponding author upon reasonable request.
