# Supplementary material for: Minimally invasive colonoscopy treatment of inflammatory fibroid polyps in the terminal ileum
Source: Sci Rep. 2023 Mar 26;13:4929. doi: 10.1038/s41598-023-31719-0 (PMC10040406; doi:10.1038/s41598-023-31719-0)
Supplement: Supplementary file 1 — Supplementary Legends. [file 41598_2023_31719_MOESM1_ESM.docx]

**Video Caption**

Video 1:

**The Endoscopic Procedure:** A normal saline solution containing methylene blue, Hemocoagulase Bothrops Atrox, and hyaluronic acid was injected into the submucosa at the distal side of the lesion. To create the submucosal space, a mucosa-cutting knife was used to incise the mucosa at the proximal side of the lesion. The incision was extended along the muscularis propria on both sides of the lesion until the tumor was completely exposed. The submucosal blood vessels were severed with a mucosa-cutting knife or thermal forceps. After the tumor was completely dissected, the mucosa at the proximal side of the lesion was severed using a mucosa-cutting knife. The wound was closed after hemostasis with thermal forceps (in this case, a hemoclip combined with nylon suture), and the specimens were collected.
